# Azithromycin Attenuates Bleomycin-Induced Pulmonary Fibrosis Partly by Inhibiting the Expression of LOX and LOXL-2

**DOI:** 10.3389/fphar.2021.709819

**Published:** 2021-11-05

**Authors:** Xiang Tong, Shijie Zhang, Dongguang Wang, Li Zhang, Jizheng Huang, Tianli Zhang, Hong Fan

**Affiliations:** Department of Respiratory Medicine and Critical Care Medicine, West China Hospital/West China School of Medicine, Sichuan University, Chengdu, China

**Keywords:** pulmonary fibrosis, azithromycin, LOX, JNK pathway, inflammation

## Abstract

Pulmonary fibrosis (PF) is a chronic and progressive process of tissue repair. Azithromycin (AZM) may be beneficial for the treatment of PF because AZM has anti-inflammatory and immune regulatory roles and inhibits remodeling, but the mechanism is not entirely clear. In this study, we established a mouse PF model induced by bleomycin (BLM) and primary mouse lung fibroblasts stimulated by transforming growth factor (TGF)-β1 to explore the possible mechanisms of AZM in PF. Results showed that AZM reduces mortality and lung inflammation and attenuates BLM-induced PF in mice. AZM effectively reduced the expression of α-smooth muscle actin (SMA) and type I collagen. Meanwhile, expression of lysyl oxidase (LOX) and lysyl oxidase-like protein (LOXL)-2 in the lung tissue of mice after AZM treatment was significantly lower than in the BLM group. In addition, this study found that AZM significantly inhibits the TGF-β1/Smad and JNK/c-Jun signaling pathways *in vivo*, and expression of a-SMA, type I collagen, LOX and LOXL-2 in the lung tissue of mice treated with AZM was significantly lower than that in the BLM group. *In vitro*, AZM also effectively inhibited type I collagen, LOX, LOXL-2 and JNK-c-Jun signaling pathways in TGF-β1-stimulated primary mouse fibroblasts, and this effect was similar to that of a JNK-specific inhibitor (SP600125). In conclusion, AZM effectively attenuated BLM-induced PF in mice, which may play a role by partially inhibiting the JNK/c-Jun and TGF-β1/Smad signaling pathways and reducing production of LOX and LOXL2.

## 1 Introduction

Pulmonary fibrosis (PF) can occur in a variety of clinical conditions and is a chronic and progressive tissue repair response process that leads to irreversible scarring and remodeling of the lung (Noble et al., 2012). Many factors, including respiratory virus infection, connective tissue disease (CTD), environmental and occupational exposure, therapy (such as radiotherapy and immunotherapy), diabetes, gastroesophageal reflux and so on, can cause and maintain fibrosis (Noble et al., 2012; Sgalla et al., 2019). Usually, the type of PF with known etiology is called secondary PF. However, PF can also occur in the absence of any known causes, known as idiopathic pulmonary fibrosis (IPF). Although IPF is considered rare, the incidence rate of IPF is increasing over time, and the prognosis is very poor. In IPF, the median survival time from diagnosis was 2–4 years, which is similar to that of many malignant tumors (Olson et al., 2007; Noble et al., 2012). However, in secondary PF, the prognosis of PF may be different with distinct etiologies. For example, viral pneumonia, including COVID-19 and severe acute respiratory syndrome (SARS), could lead to PF, but most of these patients gradually recover in the later stage (Naik and Moore, 2010; Mineo et al., 2012; George et al., 2020). In contrast, CTD combined with PF may lead to a significant increase in mortality (Cottin et al., 2018; Spagnolo et al., 2021).

The most important part of the pathophysiological mechanism of PF is the accumulation and remodeling of extracellular matrix (ECM) in the lung (Kulkarni et al., 2016; Upagupta et al., 2018). In addition to the abnormal proliferation of fibroblasts and their excessive secretion of ECM, PF is closely related to changes in ECM components or traits caused by posttranslational modifications, such as glycosylation, transglutamination, and cross linking (Upagupta et al., 2018). Previous studies have shown that lysyl oxidase (LOX) and its four lysyl oxidase-like proteins (LOXL1-4) play a key role in cross linking of the ECM (Philp et al., 2018; Chen et al., 2019; Vallet and Ricard-Blum, 2019; Nguyen et al., 2021). The LOX protein family consists of copper amine oxidases characterized by a highly conserved catalytic domain, a lysine tyrosine quinone cofactor and a conserved copper binding site. Its primary function is to catalyze covalent cross-linking of ECM protein collagens and elastin, which can lead to changes in the stiffness and mechanical properties of the ECM (Vallet and Ricard-Blum, 2019). Tjin et al. found that LOXL1 and LOXL2 expression was significantly increased in the lung tissue of IPF, and inhibition of LOX reduced PF (Tjin et al., 2017). Regulation of the LOX protein family involves a series of signaling pathways, including transforming growth factor β (TGF-β), platelet-derived growth factor, epidermal growth factor receptor and inflammatory pathways (Cheng et al., 2014; Laczko and Csiszar, 2020). In recent years, the LOX protein family has been recognized as a potential target for the treatment of PF (Chen et al., 2019).

Azithromycin (AZM) is a broad-spectrum antibacterial macrolide drug that has attracted increasing attention due to its immunomodulatory effect in addition to its antibacterial activity. Macrolide antibiotics have been used as immunomodulatory drugs in chronic obstructive pulmonary disease (COPD), asthma, and bronchiectasis (Yamaya et al., 2012; Tong et al., 2015; Kelly et al., 2018), although their use is still controversial. Wuyts et al. found that AZM attenuated bleomycin (BLM)-induced PF, but the mechanisms whereby this occurred were unclear (Wuyts et al., 2010). Recently, some observational clinical studies have found that AZM reduces the mortality of acute exacerbation and the hospitalization rate in IPF patients (Kawamura et al., 2017; Macaluso et al., 2019). Tsubouchi et al. found that AZM inhibited NADPH oxidase 4 by promoting proteasome degradation, thereby inhibiting myofibroblast differentiation and the development of lung fibrosis (Tsubouchi et al., 2017). Additionally, a recent study showed that AZM promotes the apoptosis of fibroblasts in IPF to exert an antifibrotic effect (Krempaska et al., 2020). In general, these studies suggest that AZM may play a beneficial role in PF, but the specific regulatory mechanisms still need to be further explored.

In addition to the classical TGF-β signaling pathway, the JNK/c-Jun signaling pathway is a member of the mitogen-activated protein kinase (MAPK) superfamily, which is involved in cell proliferation and differentiation, cytoskeleton construction, apoptosis, and inflammation and in the differentiation and apoptosis of fibroblasts (Davis, 2000; Yeap et al., 2010). In our study, we hypothesized that AZM inhibits LOX and LOXL-2 expression partly through the TGF-β1/Smad and JNK/c-Jun signaling pathways, thereby attenuating the degree of PF. We explored this hypothesis through BLM-induced mouse and TGF-β1-stimulated mouse primary fibroblast models.

## 2 Materials and Methods

### 2.1 Materials

BLM and the JNK inhibitor (SP600125) were obtained from Selleck China Inc. (Shanghai, China). TGF-β1 was purchased from PeproTech China Inc. (Suzhou, China). Azithromycin was obtained from Sigma-Aldrich Inc. (Shanghai, China). The primary antibodies we used are as follows: anti-vimentin (Proteintech, 60330-1-Ig), anti-alpha-smooth muscle actin (α-SMA) (Proteintech, 14395-1-AP), anti-Collagen 1 (Proteintech, 14695-1-AP), anti-LOX (Proteintech, 17958-1-AP), anti-LOXL2 (Abcam, 96233), anti-TGF-β1 (Proteintech, 21898-1-AP), anti-Smad2 (Cell Signaling Technology, 5339), anti-Smad3 (Cell Signaling Technology, 9523), anti-phospho (P)-smad2 (Cell Signaling Technology, 3108), anti-P-smad3 (Cell Signaling Technology, 9520), anti-JNK (Proteintech, 66210-1-Ig), anti-c-Jun (Proteintech, 66313-1-Ig), anti-P-JNK (Proteintech, 80024-1-RR), anti-P-cJun (Proteintech, 28891-1-AP), anti-α-tubulin (Proteintech, 66031-1-Ig), and anti-GAPDH (Proteintech, 60004-1-Ig). The dilution ratio of all antibodies was 1:1000.

### 2.2 Mouse Models and Treatment

Male C57BL/6 mice (21.3 ± 0.5 g), 7–8 weeks of age, were supplied by Beijing HFK Bioscience Co. Ltd. (Beijing, China). The mouse model of BLM-induced PF was based on previous literature published by our team (Zhang et al., 2020). AZM was dissolved in ethanol and diluted in normal saline. AZM was administered intraperitoneally at a dose of 50 mg/kg/day (the dose refers to the “toxicology” section of AZM drug instructions, which is equivalent to a dose of 500 mg/day in adults). The dose of AZM used in our study was consistent with that used in a previous asthma study (Beigelman et al., 2009). A total of 48 mice were divided into the following four groups: control: mice were intratracheally atomized with 50 μl of normal saline on day 0 and intraperitoneally injected with 100 μl of normal saline on day 7 for 3 weeks; BLM: mice were intratracheally atomized with 50 μl of BLM on day 0; BLM + AZM: mice were intratracheally atomized with 50 μl of BLM on day 0 and intraperitoneally injected with 100 μl of AZM on day 7 for 3 weeks; and AZM: mice were intratracheally atomized with 50 μl of normal saline on day 0, and intraperitoneal injection of 100 μl AZM was performed on day 7, lasting for 3 weeks. On the 28th day, mice were sacrificed by intraperitoneal injection of excessive sodium pentobarbital. All animals received care in accordance with the recommendations of the National Institutes of Health Guide for Care and Use of Laboratory Animals, and this experimental protocol was approved by the Committee on the Ethics of Animal Experiments of West China Hospital, Sichuan University (No. 2019022A).

### 2.3 Micro-CT Scanning

Referring to the method provided in previous literature (van Deel et al., 2016; Ruscitti et al., 2017), on the 28th day, all mice were scanned by microcomputed tomography (micro-CT). After mice were anesthetized with isoflurane, mouse lung imaging was conducted by a Quantum GX Micro-CT scanner (PerkinElmer, Inc., Waltham, MA) using the cardiorespiratory gated technique (Ruscitti et al., 2017). Images were obtained with an X-ray tube set to 90 kVp and 160 μA, projection radiographs were taken during the whole 360° gantry rotation, and the total scanning time was 4.5 min (Ruscitti et al., 2017).

### 2.4 Histological Analysis and Immunohistochemistry

Lung tissues were fixed in 4% formalin buffer, embedded in paraffin, and cut into 4 μm thick tissue sections. The sections were stained with hematoxylin-eosin (HE) and Masson’s trichrome staining. According to previous literature, the Ashcroft scoring system was used to assess the level of fibrosis (Ashcroft et al., 1988). Immunohistochemistry was used to evaluate the expression level of type I collagen in lung tissue. After the sections were dewaxed and rehydrated, endogenous peroxidase activity was inactivated with 3% H_2_O_2_. The sections were blocked in 5% bovine serum albumin and incubated with the anti-collagen I primary antibody at a dilution of 1:200. Then, the sections were incubated with the secondary antibody at room temperature and developed with diaminobenzidine for observation.

### 2.5 Cell Culture

According to the study published by Edelman et al., primary lung fibroblasts were isolated by the crawl out method (Edelman and Redente, 2018). Purified cells were identified by vimentin immunofluorescence using previously reported methods (Donaldson, 2015). Cells were seeded in Dulbecco’s modified Eagle medium (DMEM) containing 10% fetal bovine serum (Gibco, USA) and 1% penicillin-streptomycin (HyClone, USA) and cultured in a 37°C incubator with a humidified 5% CO_2_ atmosphere. Cell experiments were divided into 6 groups: control, TGF-β1, TGF-β1+AZM, TGF-β1+JNK inhibitor, AZM, and JNK inhibitor. To establish a cell model, primary lung fibroblasts were stimulated with 5 ng/ml TGF-β1 for 48 h as in the TGF-β1 group. Six hours before stimulation with TGF-β1, 10 μg/ml AZM was added to the culture medium as the intervention group (TGF-β1+AZM group). In addition, to verify whether AZM partially regulated the JNK/c-Jun signaling pathway involved in LOX and LOXL-2 expression, we added a JNK1 inhibitor (20 μM) 6 h before TGF-β1 stimulation (TGF-β1+JNK inhibitor group). Primary cells were used after no more than the fifth generation.

### 2.6 Real-Time PCR Analysis

Total RNA was extracted from lung tissue using TRIzol reagent (Invitrogen, USA) and reverse transcribed into complementary DNA (cDNA) according to the instructions of the PrimeScript™ RT reagent kit (Takara, Japan). iTaq Universal SYBR Green Supermix (Bio-Rad, United States) was used for real-time PCR (RT-PCR) to determine mRNA levels of α-SMA, collagen I, LOX, LOXL-2, and GAPDH. Table 1 shows the primer sequences, relative gene expression levels were normalized to GAPDH and calculated using the 2^−ΔΔCt^ method.

**TABLE 1:** Primers for quantitative RT-PCR.

Primer name	Sequence (5′ to 3′)
M-collagen I-F	AAG​AAG​CAC​GTC​TGG​TTT​GGA​G
M-collagen I-R	GGT​CCA​TGT​AGG​CTA​CGC​TGT​T
M-α-SMA-F	GTA​CCA​CCA​TGT​ACC​CAG​GC
M-α-SMA-R	GAA​GGT​AGA​CAG​CGA​AGC​CA
M-LOX-F	ACT​TCT​TAC​CAA​GCC​GCC​CT
M-LOX-R	TGG​CAT​CAA​GCA​GGT​CAT​AGT​G
M-LOXL2-F	GGA​GCT​TTT​CTT​CTG​GGC​AAC​C
M-LOXL2-R	TAC​TCA​GGG​TAC​TGG​AGC​TGG
M-GAPDH-F	CCT​CGT​CCC​GTA​GAC​AAA​ATG
M-GAPDH-R	TGA​GGT​CAA​TGA​AGG​GGT​CGT

RT-PCR, real-time polymerase chain reaction; SMA, smooth muscle actin; LOX, lysyl oxidase; LOXL2, lysyl oxidase-like protein-2.

### 2.7 Western Blot Analysis

Lung tissues or cells were fully lysed in RIPA buffer (Beyotime, China) containing a fresh mixture of protease and phosphatase inhibitors (MedChemExpress, United States).

The entire process was performed at 4°C. After centrifugation at 12000 r/min for 20 min, the supernatant was added to 5× protein sample loading buffers (Epizyme, China) and boiled for 10 min. A BCA protein kit (Thermo, USA) was used to determine protein concentrations. Denatured proteins were separated by 10% SDS-PAGE (Epizyme, China) and then transferred onto methanol-activated PVDF membranes (Millipore, USA) at a constant current of 400 mA. After blocking with 5% skim milk for 1 h, membranes were incubated with different primary antibodies overnight at 4°C. After washing the PVDF membrane several times, it was incubated with the appropriate secondary antibody (1:2,000) for 1 h at room temperature. Subsequently, ECL (GE Healthcare, United Kingdom) was used to visualize protein expression, and ImageJ software was used to analyze the band intensities.

### 2.8 Statistical Analysis

Statistical analysis was performed using GraphPad Prism Version 9.0 (GraphPad software, USA). All raw data are shown as the mean ± standard deviation. One-way ANOVA tests were used for analyzing differences, and Tukey’s multiple comparison test was used to compare multiple groups. The Kaplan-Meier method was used to draw the survival curve of each group. A *p*-value less than 0.05 was considered statistically significant.

## 3 Results

### 3.1 Azithromycin Attenuates Bleomycin-Induced Pulmonary Fibrosis in Mice

**FIGURE 1:**
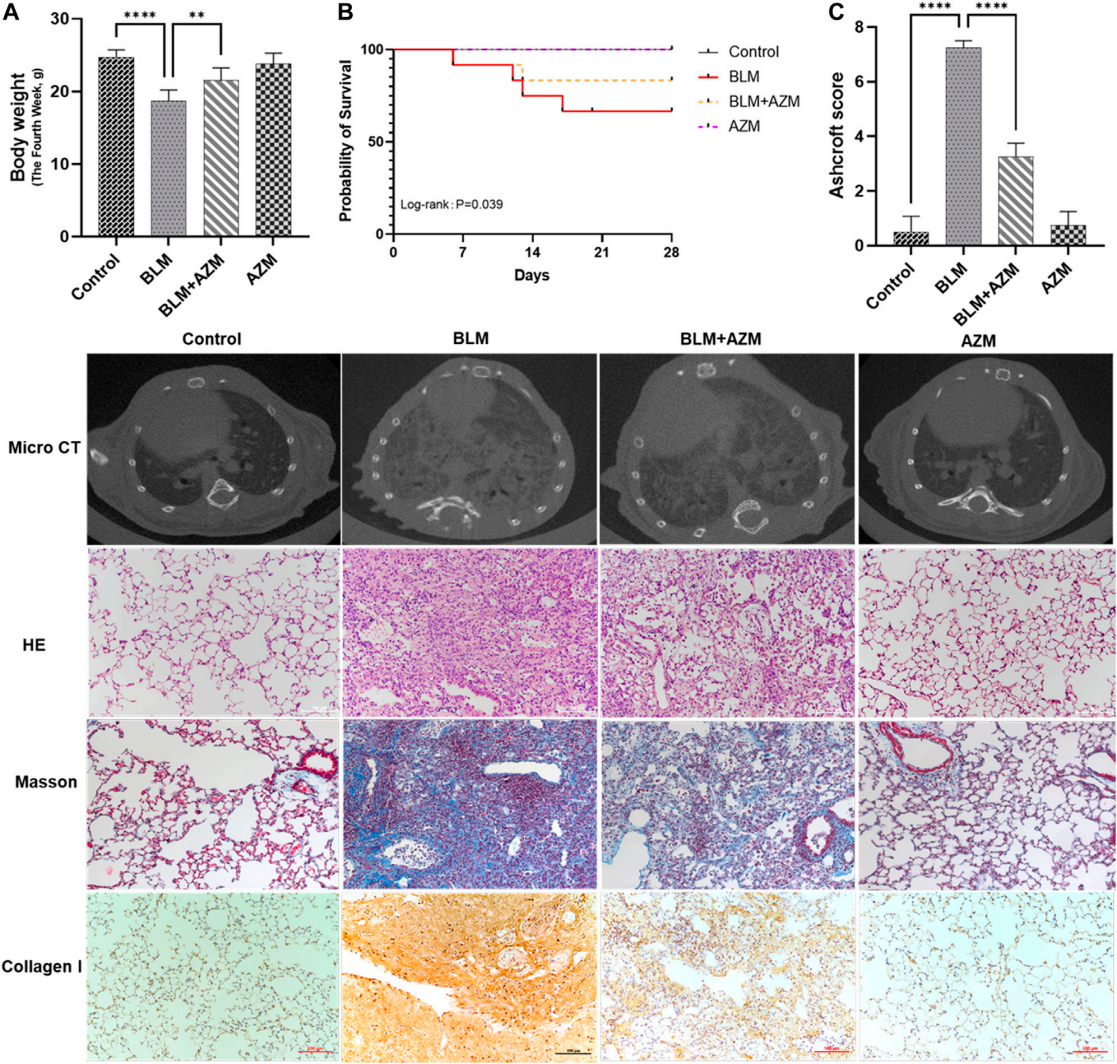
Azithromycin (AZM) attenuated bleomycin (BLM)-induced pulmonary fibrosis in mice. **(A)** Mice body weights were measured in Day 28. **(B)** Survival rate of mice in different groups. **(C)** Ashcroft score for the four groups were based on HE staining. The other images were micro CT results, HE staining, Masson staining, and collagen I staining in different groups (×200 magnification; scale bars = 100 μm). Data were presented as the means ± SD. **p < 0.01, ****p < 0.0001.

After a single intratracheal atomization of bleomycin (BLM), compared to the control group, the weight of mice in the BLM group was significantly reduced on day 28, and the mortality rate was 41.7% in the BLM group, which was significantly reversed (16.7%) after treatment with AZM (Figures 1A,B). There was no significant difference between the control group and the AZM group (Figures 1A,B). Microcomputed tomography (micro-CT) results showed that after a single dose of BLM intratracheal atomization, the lung structure of mice was destroyed, and imaging features of PF, such as grid shadow, strip shadow, honeycomb lung, and interstitial thickening, appeared in both lungs (Figure 1).

As shown in Figure 1, hematoxylin-eosin (HE) staining showed that in the BLM group, the alveolar septum was thickened, the alveolar structure was destroyed, and a large number of red blood cells and inflammatory cells infiltrated the alveolar and lung interstitium, while the alveolar structure in the control and AZM groups was normal. Masson staining revealed a large amount of collagen deposition in the lung tissue of the BLM group compared to the control group. AZM treatment significantly reduced inflammatory cell infiltration and collagen deposition and improved alveolar structure.

The fibrosis score of the BLM group was significantly higher than that of the control group, while the fibrosis score of the AZM treatment group was significantly reduced, suggesting that AZM effectively reduces BLM-induced pulmonary fibrosis (Figure 1C, *p* < 0.001).

### 3.2 Azithromycin Inhibits Expression of LOX and LOXL-2 in Mice With Bleomycin-Induced Pulmonary Fibrosis

**FIGURE 2:**
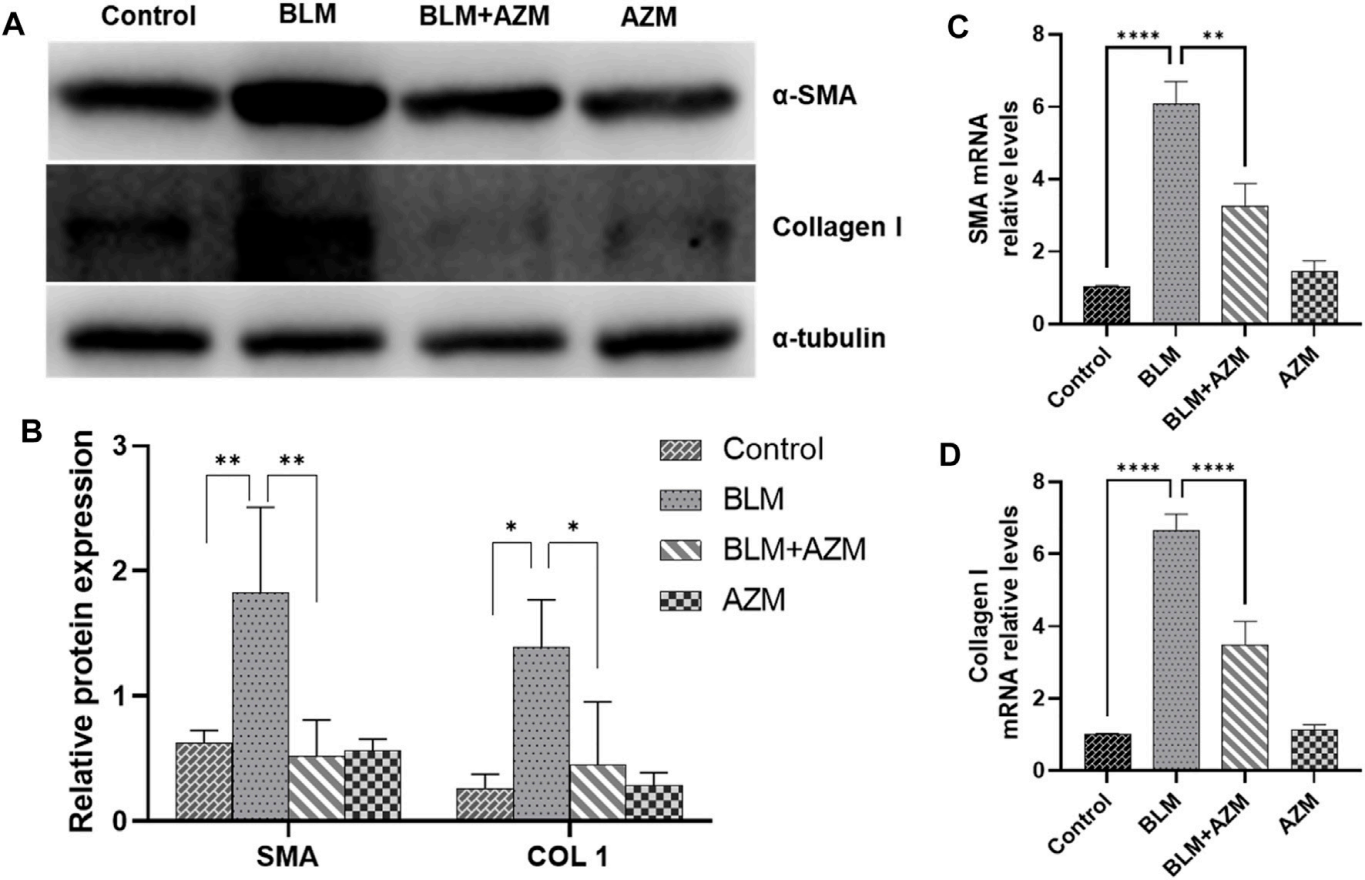
AZM reduced the expression of alpha-smooth muscle actin (α-SMA) and Collagen 1 (COL 1) in lung tissue of mice with pulmonary fibrosis. **(A,B)** The protein expression of α-SMA and COL 1 was measured in each group by Western blot. **(C,D)** The gene expression of α-SMA and COL 1 was measured in each group by RT-PCR. Data were presented as the means ± SD (*n* ≥ 3). *p < 0.05, **p < 0.01, ****p < 0.0001.

**FIGURE 3:**
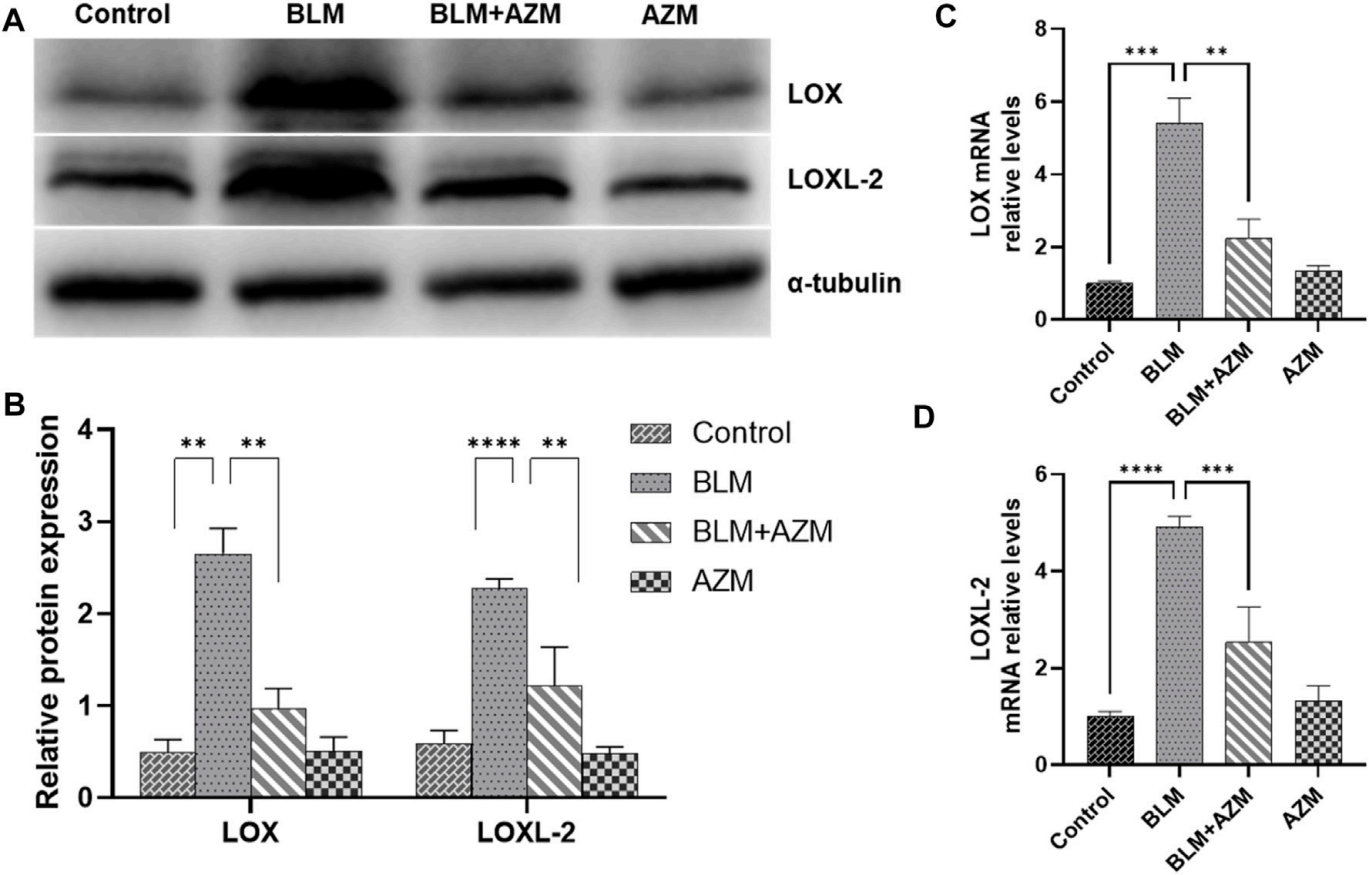
AZM reduced the expression of lysyl oxidase (LOX) and lysyl oxidase-like protein (LOXL2) in lung tissue of mice with pulmonary fibrosis. **(A,B)** The protein relative expression of LOX and LOXL2 was measured in each group by Western blot. **(C,D)** The gene expression of LOX and LOXL2 was measured in each group by RT-PCR. Data were presented as the means ± SD (*n* ≥ 3). **p < 0.01, ***p < 0.001, ****p < 0.0001.

LOX and LOXL-2 were found to be closely related to PF and interacted with the TGF-β and JNK signaling pathways (Sethi et al., 2011; Chien et al., 2014; Wei et al., 2017; Wu et al., 2018). As shown in Figure 2, western blot and Real-Time-polymerase chain reaction (RT-PCR) showed that protein and gene expression levels of α-smooth muscle actin (a-SMA) and type I collagen in the BLM group were significantly higher than those in the control group, and AZM effectively reduced expression levels of a-SMA and type I collagen. In addition, expression of LOX and LOXL-2 in the BLM group was significantly upregulated compared to the control group. After AZM intervention, expression of LOX and LOXL-2 in the BLM group was significantly lower than in the BLM group, exhibiting no difference from the control group (Figure 3).

### 3.3 Azithromycin Inhibits the Activities of TGF-β/Smad and JNK/C-Jun Signaling Pathways in Mouse Pulmonary Fibrosis

**FIGURE 4:**
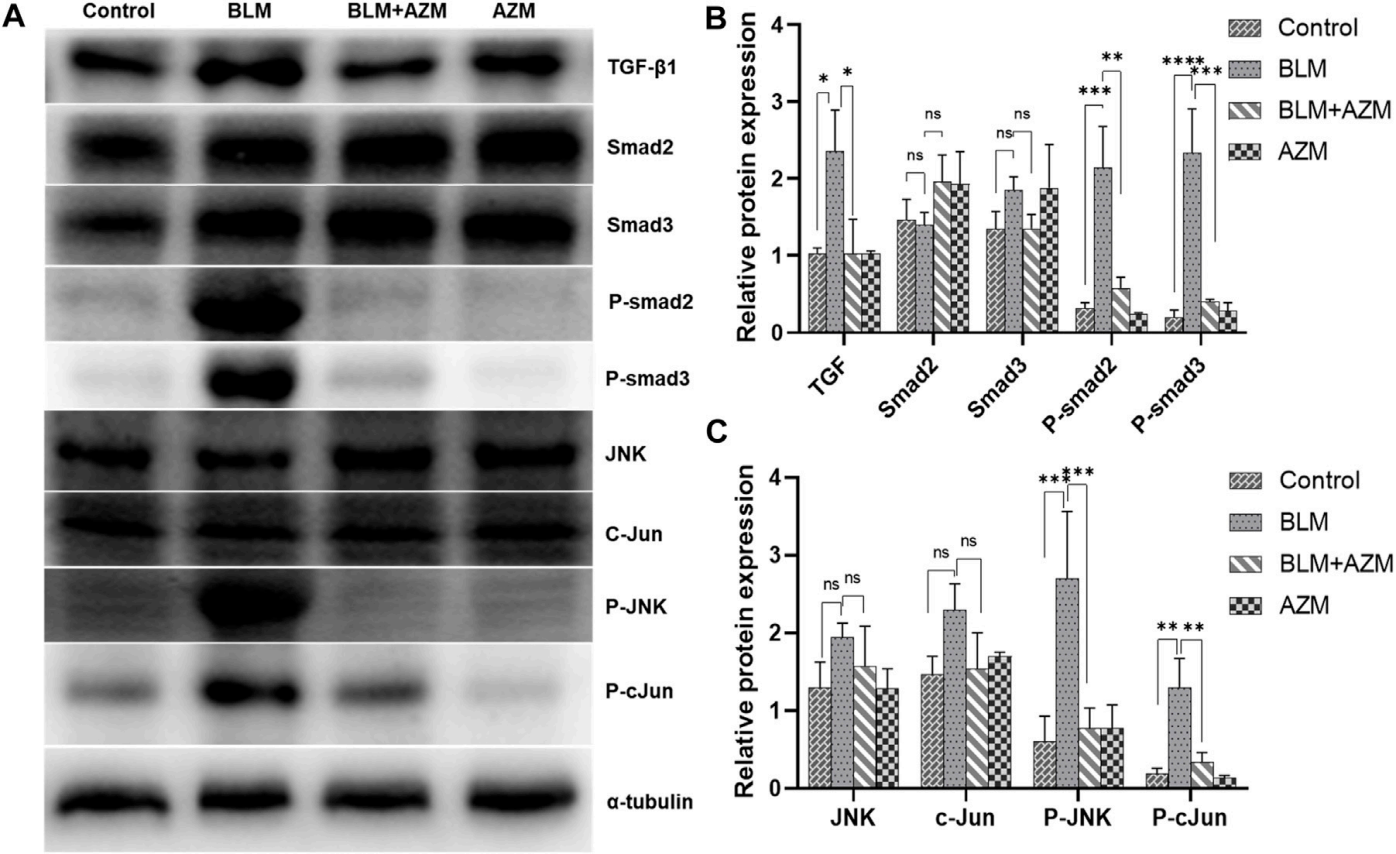
AZM suppressed the TGF-β1/Smad and JNK/c-Jun signaling pathway in BLM-induced mice. **(A)** Western blotting bands of TGF-β1/Smad and JNK/c-Jun pathway proteins in lung tissue of mice in different groups. **(B,C)** The protein relative expression of TGF-β1/Smad and JNK/c-Jun pathway proteins was measured in each group by Western blot. Data were presented as the means ± SD (*n* ≥ 3). **p < 0.01, ***p < 0.001, ****p < 0.0001, ns, non-significant.

The TGF-β signaling pathway is the most important regulatory pathway in pulmonary fibrosis. The activated TGF-β signaling pathway directly upregulates gene expression of the ECM and stimulates expression of many proinflammatory and fibrosis cytokines, such as interleukin, tumor necrosis factor-α, or platelet-derived growth factor, to further enhance and maintain the fibrotic response (Kang, 2017). The results showed that AZM significantly inhibited expression of TGF-β1 and phosphorylated Smad2 and Smad3 in the lung tissue of BLM-treated mice, while total Smad2 and Smad3 protein levels did not change (Figure 4). In addition, the JNK/c-Jun signaling pathway, another important pathway in the regulation of fibrosis, has attracted much attention in recent years (Grynberg et al., 2017). The results showed that expression levels of phosphorylated JNK and phosphorylated c-Jun proteins in the BLM group were significantly increased. Total JNK and c-Jun protein levels did not change, but JNK and c-Jun mRNA levels were significantly increased. AZM inhibited expression of phosphorylated JNK and phosphorylated c-Jun in the lung tissues of BLM-treated mice (Figure 4).

### 3.4 Azithromycin Attenuates LOX and LOXL-2 Expression in Mouse Lung Fibroblasts by Partially Inhibiting the JNK/C-Jun Signaling Pathway

**FIGURE 5:**
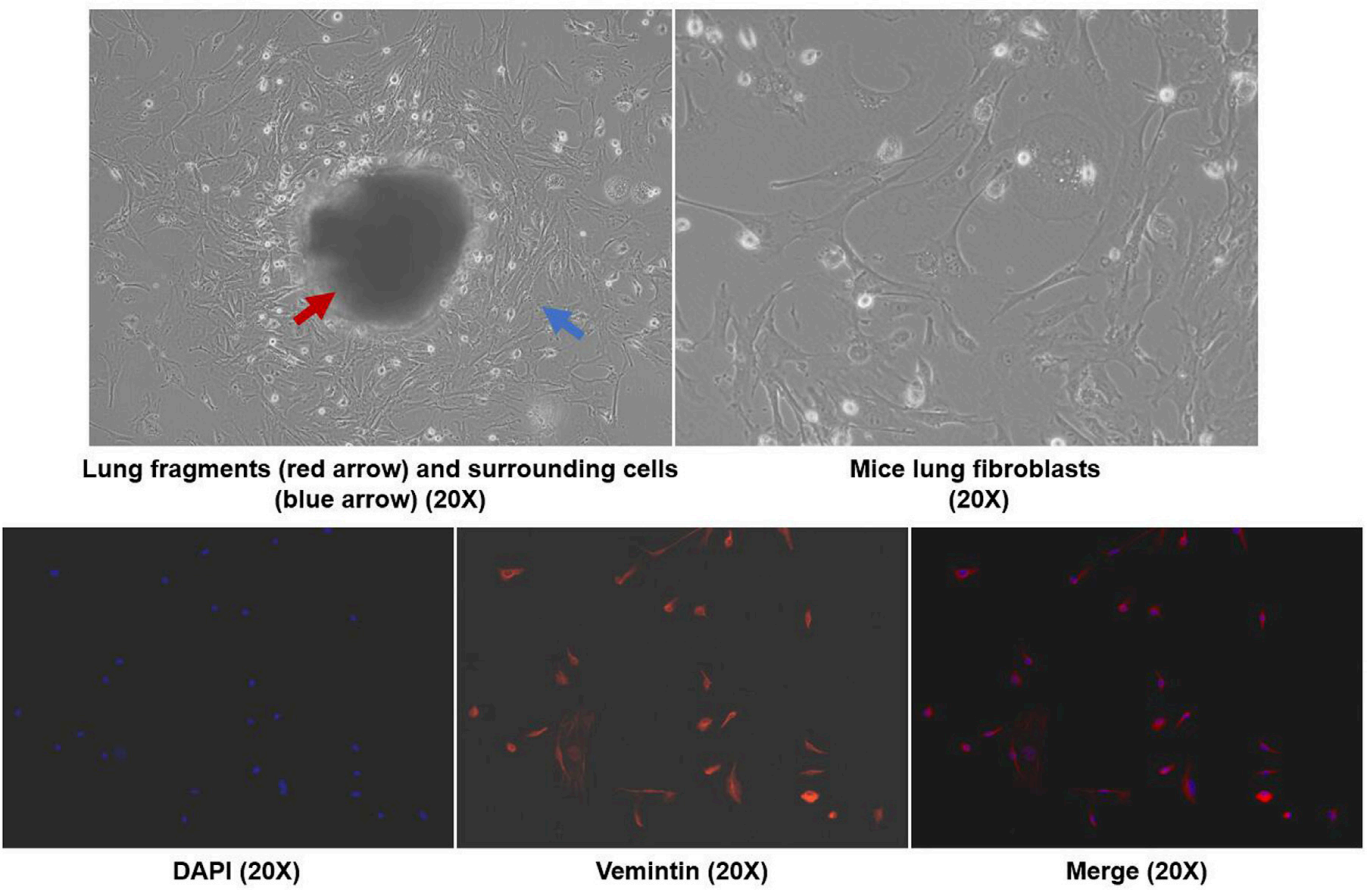
Mice primary lung fibroblasts were isolated by crawl out method, and the immunofluorescence with anti-vimentin antibody was used to identify the cell purity (× 200 magnetization).

**FIGURE 6:**
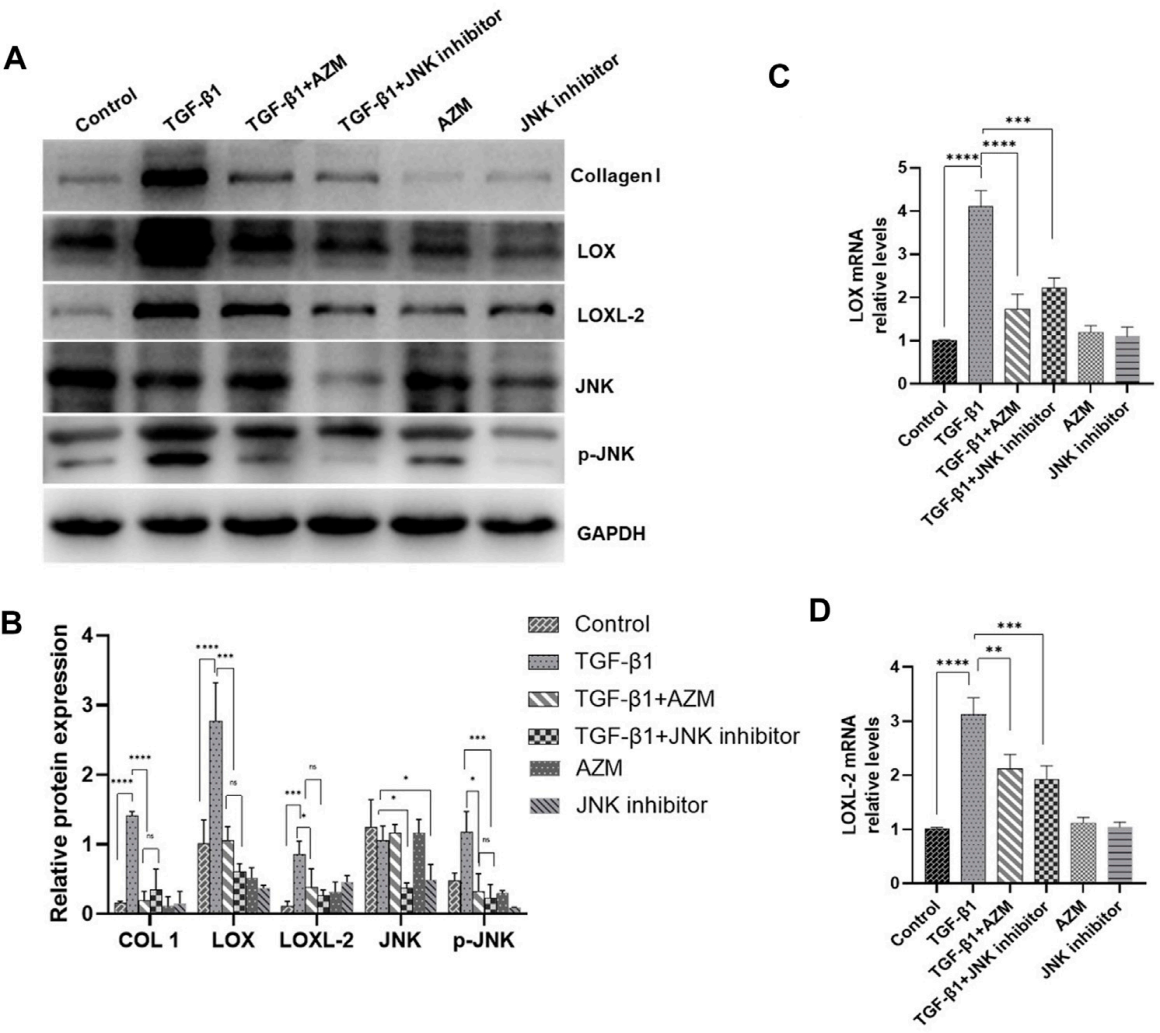
AZM and JNK1 inhibitor (SP600125) have similar effects, and reduced the expression of LOX and LOXL2 in mice primary lung fibroblasts induced by 5 μg/ml TGF-β1. **(A,B)** The protein expression of LOX, LOXL2, COL1, JNK and GAPDH was measured in each group by Western blot. **(C,D)** The gene expression of LOX and LOXL2 was measured in each group by RT-PCR. Data were presented as the means ± SD (*n* ≥ 3). *p < 0.05, **p < 0.01, ***p < 0.001, ****p < 0.0001, ns, non-significant.

As shown in Figure 5, we extracted mouse primary lung fibroblasts using previously reported research methods (Edelman and Redente, 2018) and determined the purity of these cells by fluorescence detection of vimentin. The results showed that the purity of primary lung fibroblasts was greater than 90%. According to previous literature, TGF-β1 (5 ng/ml) was used to stimulate fibroblasts to establish a cell model. LOX, LOXL-2, phosphorylated JNK and type I collagen were all significantly increased in response to TGF-β stimulation, while LOX, LOXL-2 and type I collagen levels were decreased and expression of phosphorylated JNK protein decreased simultaneously in response to AZM intervention. After blocking the JNK signaling pathway with SP600125, it was found that it had a similar effect to AZM intervention, and expression of LOX, LOXL-2, type I collagen, total JNK and phosphorylated JNK was significantly reduced (Figure 6). Therefore, these results preliminarily suggest that AZM may inhibit expression of LOX and LOXL-2 in fibroblasts, partly through the JNK/c-Jun signaling pathway.

## 4 Discussion

Macrolide antibiotics, as anti-inflammatory and immunomodulatory agents, have become potential candidates for the treatment of PF (Krempaska et al., 2020). Our study revealed that AZM effectively reduced expression levels of α-SMA and type I collagen in BLM-induced PF in mice (Figure 1). *In vitro*, the study found that AZM also effectively inhibited the expression of type I collagen in mouse lung fibroblasts stimulated by TGF-β1 (Figure 6), which is consistent with results previously reported by Wuyts et al. (Wuyts et al., 2010). In addition, AZM could play a key role in reducing tissue remodeling through a variety of mechanisms, such as inhibiting airway epithelial cell apoptosis and epithelial-mesenchymal transition (Liu et al., 2017; Pu et al., 2018). A series of small sample retrospective studies suggested that macrolide antibiotics may improve the prognosis of patients with interstitial lung disease (ILD) (Kawamura et al., 2017; Nagasawa et al., 2021). We conducted a meta-analysis (data unpublished), and found that AZM effectively reduced the mortality of patients with acute exacerbation of ILD, and reduced the incidence of mechanical ventilation in patients.

The LOX protein family, a cross-linked enzyme of the ECM, plays a key role in ECM remodeling and modification, regulating the development of fibrosis (Aumiller et al., 2017; Bellaye et al., 2018; Guo et al., 2020). Our study showed that the expressions of LOX and LOXL-2 were significantly increased in BLM-induced PF in mice (Figure 3). To our knowledge, this study was the first time to show that AZM effectively inhibited expression of LOX and LOXL2 in BLM-induced PF in mice. *In vitro*, our study revealed that AZM also inhibited the expression of LOX and LOXL2 in TGF-β1-stimulated mouse primary fibroblasts, and this inhibitory effect is similar to the use of JNK specific inhibitors (Figure 6). Aumiller et al. found that expression of LOX and LOXL2 was significantly increased in IPF patients, mouse models and cell models of pulmonary fibrosis (Aumiller et al., 2017). Chien et al. found that higher serum LOXL2 levels was associated with increased risk for IPF disease progression (Chien et al., 2014). In animal study, LOX inhibitors (β-Aminopropiononitrile) could reduce myocardial fibrosis and alleviating myocardial hypertrophy (Martínez-Martínez et al., 2016). Guo et al. (2020) found that triptolide prevents nuclear translocation of NF-κB and DNA binding, effectively reducing the expression of LOX and alleviating the degree of radiation-induced PF in mice. Ikenaga et al. found that selective targeting of LOXL2 inhibits the progression of liver fibrosis and accelerates its reversion (Ikenaga et al., 2017). In a phase II clinical trial (NCT01769196), simtuzumab, a monoclonal antibody against LOXL2, did not improve progression-free survival in IPF patients (Raghu et al., 2017). However, the failure of the clinical trial may be attributed to lack of tissue penetration of the drug in human IPF lung (Meyer, 2017). Since AZM is highly enriched in lung tissues (Parnham et al., 2014), and it could effectively reduce the expression of LOX and LOXL-2, it may have great potential application value for the treatment of PF in the future.

**FIGURE 7:**
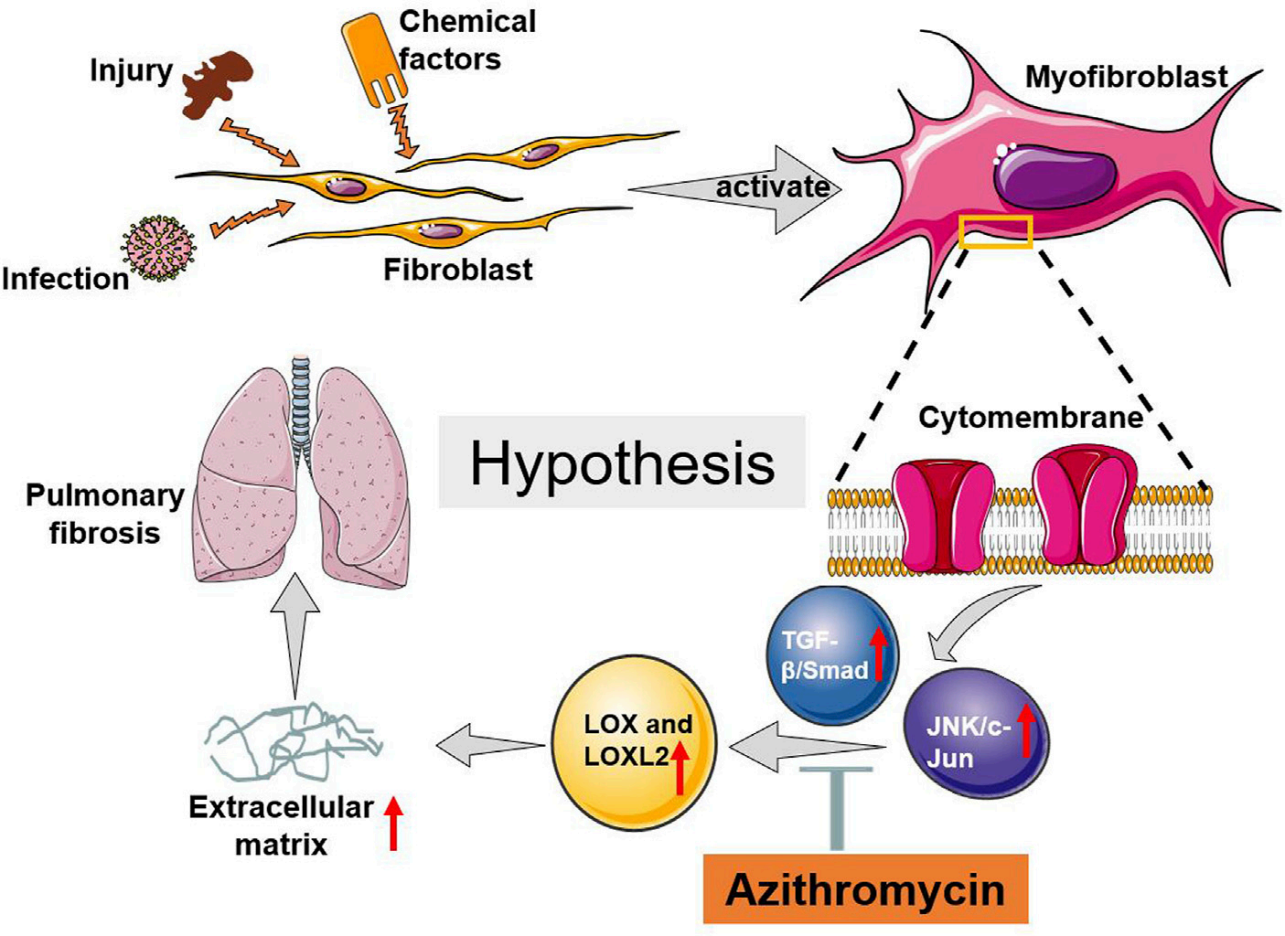
The possible potential mechanism of AZM attenuated BLM induced pulmonary fibrosis in mice.

Additionally, our results demonstrated that AZM significantly inhibited the TGF-β signaling pathway (Figure 4). In fibrotic disease, the TGF-β signaling pathway is primarily involved in regulating fibroblasts and EMT activation, promoting ECM production, maintaining fibroblast activity, and inhibiting metalloproteinases (Biernacka et al., 2011; Hu et al., 2018). In addition, TGF-β is widely involved in inflammation and immune regulation, which is also a crucial process in fibrosis (Meng et al., 2014; Hu et al., 2018). Ruan et al. found that AZM effectively inhibited TGF-β1 signaling, weaken the activation and differentiation of lung fibroblasts (Ruan et al., 2021). Previous studies have shown that TGF-β1 can significantly upregulate LOX mRNA and protein levels in fibroblasts and epithelial cells during fibrosis (Remst et al., 2014). Other studies have suggested that LOX regulates TGF-β through a feedback loop, which plays a role in skeletal muscle development and IPF (Remst et al., 2014). In addition, direct interaction between LOX and TGF reduced TGF-stimulated Smad3 activation (Remst et al., 2014). Our study suggested that the levels of LOX and LOXL-2 increased in parallel with the level of TGF-β1 in BLM group, and AZM could inhibit the expression of LOX and TGF-β1 (Figure 4). Therefore, we speculate that AZM reduces expression of LOX by inhibiting the TGF-β signaling pathway to attenuate the degree of PF (Figure 7).

The JNK/c-Jun signaling pathway is a member of the mitogen-activated protein kinase (MAPK) superfamily, which plays an important role in cellular differentiation, apoptosis, stress response, inflammation and the occurrence and development of many human diseases (Bode and Dong, 2007). In a clinical study (NCT01203943), a JNK inhibitor (CC-930) effectively attenuated airway remodeling, reduced the production of pulmonary fibrosis markers, and improved lung function (van der Velden et al., 2016). However, the interaction between the JNK/c-Jun signaling pathway and the LOX protein family is still not well explored in fibrotic diseases. In the current study, we found that AZM effectively inhibited the JNK/c-Jun signaling pathway and simultaneously inhibited expression of LOX and LOXL2 (Figure 4). At the same time, we found that JNK pathway-specific inhibitors effectively inhibited expression of LOX and LOXL, attenuating the degree of PF (Figure 6). Our results were similar to the previous studies. A recent study suggested that AZM inhibits the MAPK/JNK signaling pathway in a human monocytic cell line (THP-1) induced by LPS (Kuo et al., 2019). Hiwatashi et al. (2011) found that AZM inhibits the proliferation of peripheral blood mononuclear cells by suppressing the activity of JNK and ERK. Based on the above findings, we speculated that AZM partially inhibits the JNK/c-Jun signaling pathway, downregulates expression of LOX and LOXL-2 levels, reduces the production of ECM, and ultimately attenuates PF (Figure 7).

Although our study revealed that AZM could attenuate PF by inhibiting the expression of LOX and LOXL-2, the regulatory mechanisms need to be further verified (e.g. using LOX inhibitors or knockdown mice). As we all know, a large number of cells, cytokines, enzymes, and signal pathways are involved in the regulation of ECM, but our study failed to explore other important factors (e.g. matrix metalloproteinases, MMPs) in the regulation of ECM. We will conduct in-depth and comprehensive study on the regulation mechanisms of ECM in the future, such as exploring the interaction between LOX and MMPs. In addition, more rigorous clinical studies or real-world studies need to be designed to accurately evaluate the value of AZM in the treatment of patients with PF.

In summary, AZM effectively attenuated BLM-induced PF in mice, which may occur by partially suppressing the JNK/c-Jun and TGF-β1/Smad signaling pathways and reducing LOX and LOXL2 production (Figure 7).

## Data Availability

The raw data supporting the conclusions of this article will be made available by the authors, without undue reservation.
